# Prospective study of autism phenomenology and the behavioural phenotype of Phelan–McDermid syndrome: comparison to fragile X syndrome, Down syndrome and idiopathic autism spectrum disorder

**DOI:** 10.1186/s11689-017-9217-6

**Published:** 2017-11-10

**Authors:** Caroline Richards, Laurie Powis, Jo Moss, Christopher Stinton, Lisa Nelson, Christopher Oliver

**Affiliations:** 1 Cerebra Centre for Neurodevelopmental Disorders, School of Psychology, University of Birmingham, Birmingham, B15 2TT UK; 2 Hertfordshire Partnership University Foundation Trust, West Community Assessment and Treatment Service, St. Paul’s, Off Allandale, Hemel Hempstead, Hertfordshire HP2 5XY UK; 3 Institute of Cognitive Neuroscience, University College London, London, WC1N 3AR UK; 4 Division of Health Sciences, Warwick Medical School, University of Warwick, Coventry, CV4 7AL UK

**Keywords:** Phelan–McDermid syndrome, Autism spectrum disorder, *SHANK3*, Behavioural phenotype, Mood, Repetitive behaviour, Impulsivity, Hyperactivity

## Abstract

**Background:**

The limited behavioural phenotype literature on Phelan–McDermid syndrome (PMS) indicates atypically high levels of activity, impulsivity and autism spectrum disorder (ASD) behaviours. Divergent profiles of ASD in PMS are also reported, with some studies demonstrating similarities to idiopathic ASD and others indicating an uneven profile of social and communication impairments and repetitive behaviours. An evaluation of the behavioural phenotype of PMS and the prevalence and phenomenology of ASD is warranted, particularly given the causal involvement of the *SHANK3* gene in the aetiology of PMS.

**Methods:**

Carers of individuals with PMS (*N* = 30; mean age = 10.55, SD = 7.08) completed questionnaires relating to impulsivity, overactivity, mood, interest and pleasure, repetitive behaviour and ASD phenomenology. These data were compared to data from matched samples of individuals with fragile X and Down syndromes and idiopathic ASD. In order to evaluate the profile of ASD phenomenology in PMS, two comparisons were made: first, including the total sample with PMS, and second, including only those who met the threshold indicative of autism on an ASD screening measure.

**Results:**

The results revealed lower mood in individuals with PMS, but no differences in impulsivity and overactivity. Compulsive and routine-driven repetitive behaviours were less common in the total sample with PMS; however, motor-based stereotyped behaviours were more common. ASD phenomenology was highly prevalent, with 87% of the sample meeting the cutoff score for ASD and 57% meeting the cutoff for autism. The profile of ASD phenomenology in the total sample with PMS differed from those with idiopathic ASD across impairments in communication and social interaction and repetitive behaviour. However, the profile of those who met the threshold for autism was commensurate to those with idiopathic ASD.

**Conclusions:**

ASD phenomenology is common within PMS. Whilst the total sample may display an atypical profile of ASD behaviour, the profile in those who met the threshold for autism was very similar to those with idiopathic ASD. These results are discussed in relation to the wider behavioural phenotype and the emerging evidence of an autism endophenotype in PMS.

## Background

Phelan–McDermid syndrome (PMS) is a micro-deletion syndrome with diagnosis based on cytogenetic, molecular cytogenetic and/or molecular evidence of loss or disruption at 22q13.3 [1]. The incidence of PMS is unknown, with under-diagnosis suspected due to the subtlety of the deletion [2]. Approximately 80% of people with PMS have de novo, simple terminal deletions, and the remaining 20% typically result from unbalanced translocations and ring chromosomes [1]. The 22q13 region contains the *SHANK3* gene: haploinsufficiency of *SHANK3* is proposed to cause the major features of PMS [3–5], and mutations in the *SHANK1*, *SHANK2* and *SHANK3* genes are associated with autism spectrum disorder [6]. Recent research has demonstrated that the *SHANK3* mutation results in neuronal changes, including increase in input resistance to excitability, some impairment in dendritic branching and decreases in synaptic transmission [7]. These changes are mechanistically linked to impairments in hyperpolarization-activated cation (I_h_) channels, with a suggestion that reduced I_h_ currents may account for some of the phenotypic characteristics observed in PMS [7]. Dysmorphic physical features associated with PMS are subtle and include hypotonia, normal to accelerated growth, long eye lashes, large ears, full brow, dolicocephaly, full cheeks, bulbous nose and pointed chin [1, 4, 8]. The most characteristic clinical features of PMS are moderate to profound intellectual disability and absent to severely delayed speech [1, 2, 8–10]. Preliminary research suggests that the physical features and severity of intellectual disability correlate with the size of the genetic deletion. However, expressive speech deficits are not associated with the size or type of deletion [8].

A number of behavioural characteristics have been reported in PMS. Hyperactivity, impulsivity and difficulties in sustaining attention have been identified; 34% of children with PMS were reported to have a diagnosis of attention deficit hyperactivity disorder (ADHD) [11], 36% of children scored above clinical cutoff for ADHD on a screening measure [12] and a high proportion of parents endorsed items indicative of impulsivity and inattention [11]. These findings suggest a potential association between PMS and ADHD phenomenology. However, few studies have employed measures designed for individuals with intellectual disabilities and none of the studies compared the results for the PMS group to contrast groups. Thus, it is unclear whether the presence of ADHD symptoms should be attributed to the behavioural phenotype of PMS, the severity of intellectual disability, age of the children assessed or the measures used. Similar threats to validity weaken results associating atypical affect with the behavioural phenotype of PMS. Cohort and case studies identified behaviours indicative of depression and psychosis/atypical bipolar disorder in PMS [11, 13]. However, given deficits in expressive language, it is unclear how internal experiences of positive symptoms of psychosis or depression have been reported and assessed. Nonetheless, given the clinical implications of mood disturbances, these findings warrant further investigation, utilising robust measures validated for individuals with intellectual disability and contrasting findings with appropriate comparison groups.

A final characteristic, frequently identified in PMS, is that of autism spectrum disorder (ASD) [2, 11, 12, 14] with a recent review suggesting that all individuals diagnosed with PMS should undergo specialist ASD assessments [15]. The putative association between ASD and PMS is of particular interest as the *SHANK3* gene is one of the many implicated in the aetiology of idiopathic ASD [3, 16, 17] with *SHANK3* deficiency associated with 0.5 to 2% of cases of ASD and intellectual disability [18]. Thus, delineation of the prevalence and phenomenology of ASD in PMS may have clinical implications for individuals with PMS *and* individuals with idiopathic ASD. Results from screening instruments have demonstrated convergent results: mean autism/pervasive developmental disorders scale scores for children on the Reiss Scales were above clinical cutoff [11]; 94% of children with PMS scored in the mild to moderate range for ASD and 67% in the severe range for ASD using the Childhood Autism Rating Scale [2]; 85% of children with PMS met the cutoff for ASD on the Social Communication Questionnaire (SCQ) and 67% met the more stringent cutoff for autism [12]. More robust evidence is found in studies employing clinical diagnostic measures of ASD. Soorya and colleagues [14] utilised both the Autism Diagnostic Observation Schedule (ADOS) [19] and Autism Diagnostic Interview-Revised (ADI-R) [20] and found that 84% of the sample with PMS met criteria for ASD and 75% met criteria for the more stringent classification of autistic disorder. However, whilst there appears to be a strong association between ASD phenomenology and PMS, no studies have employed contrast or comparison groups to evaluate whether ASD phenomenology can be identified as a specific component of the behavioural phenotype of PMS, rather than attributed to the level of intellectual disability associated with the syndrome. This is particularly important given the potential for over-estimating ASD when intellectual disability and expressive speech deficits are present [21].

Whilst there is a purportedly high prevalence of ASD phenomenology in PMS, the profile of ASD impairments in communication and social interaction and repetitive behaviour domains in the syndrome is less well described. The profile of ASD is known to vary across genetic syndromes [22]. For example, a recent meta-analysis demonstrated that ASD phenomenology is common in Cornelia de Lange and fragile X syndromes [23], and yet, detailed item-level analysis of screening [24] and diagnostic measures [25] reveal that both syndromes evidence an atypical profile of ASD. Philippe and colleagues [26] reported that whilst children with PMS attained high ADI-R scores, these only reached clinical thresholds in social interaction, play, and communication domains. They argue that the relative lack of repetitive behaviours distinguishes PMS from idiopathic ASD. However, the study was limited by not including an idiopathic ASD comparison group and relying upon visual inspection of data. Additionally, a number of sub-threshold items in the repetitive behaviour domains necessitated expressive language (e.g. delayed echolalia, verbal rituals). Interestingly, Soorya and colleagues [14] also found that interpretation of the ADI-R algorithm alone indicated that many children with PMS presented with sub-threshold levels of repetitive behaviour. However, when they included statistical analysis of all items, including a two factor algorithm of repetitive behaviour identified in research on the ADI-R, they found that repetitive and sensory-motor behaviours were present in the majority of the participants, and were similar in range to those reported in idiopathic ASD.

Finally, authors have suggested that behaviours indicative of psychopathology (psychosis and low mood) may be misinterpreted as ASD phenomenology in individuals with PMS [11]. Shaw and colleagues [11] report that some endorsed items could indicate both ASD and mental health problems, e.g. “Does not seem to listen when spoken to directly”, “Random and inappropriate speech” and “Appears confused”. Additionally, they suggest that other items such as “Maintains a rigid posture”, “Appears to be in a stupor, as if intoxicated” and “Laughs or appears angry for no apparent reason” may be more indicative of psychosis than ASD. However, it could be argued equally that these behaviours are indicative of repetitive behaviour, sensory difficulties or problems with emotional regulation, all of which are commonly reported in idiopathic ASD. Thus, there is a need to evaluate further the profile of ASD in PMS, utilising measures appropriate for individuals with intellectual disability, and with sufficient specificity and psychometric properties to allow for item-level statistical analysis. Additionally, these analyses need to be made in comparison to contrast groups, necessarily including individuals with idiopathic ASD, and ideally including groups with other genetic syndromes with known ASD profiles, in order to determine the relative position of the ASD profile in PMS.

A final point of interest is that the investigation of the profile of ASD impairments in PMS appears to have been largely driven by the hypothesised genetic links between PMS and idiopathic ASD. This has resulted in studies analysing the ASD profile of *all* participants in the PMS samples [11, 14, 26] in order to establish whether the profile in the syndrome is similar to individuals with idiopathic ASD. These data could support or weaken the hypothesised genetic *SHANK3* link. A complementary analysis approach would be to restrict analyses to those who score above threshold for autism and ASD. These data would answer a second question about whether individuals with PMS meet criteria for autism *for the same reasons* as individuals with idiopathic ASD. Answers to this question would inform discussion of the specific clinical needs for individuals with PMS who evidence ASD behaviours, thus increasing the specificity of clinical provision and interventions for individuals with PMS.

In summary, there is emerging evidence of attentional differences and differences of mood in individuals with PMS [11, 12]; however, these findings require further investigation utilising measures appropriate for individuals with intellectual disabilities, allowing for statistical comparisons with contrast groups. Additionally, there is evidence of a heightened prevalence of ASD phenomenology in PMS [2, 11, 12, 14]. The prevalence and profile of these ASD behaviours require further investigation with particular attention to the profile of repetitive behaviours in the syndrome. There is a need to delineate the profile of ASD phenomenology in PMS in contrast to individuals with idiopathic ASD, and individuals with genetic syndromes with known ASD profiles. Fragile X and Down syndromes may provide useful comparisons as they evidence divergent prevalence of ASD phenomenology (~ 30% in males with fragile X syndrome, ~ 16% in Down syndrome [23]) and well-known profiles of ASD behaviour. Contrasts between PMS and fragile X and Down syndromes will facilitate exploration of whether ASD phenomenology can be attributed to the behavioural phenotype of PMS, over and above the level of intellectual disability associated with presence of the syndrome. Comparisons between PMS and an idiopathic ASD contrast group will allow evaluation of whether the profile of impairments in PMS is commensurate to those seen in idiopathic ASD. Finally, given tentative hypotheses regarding diagnostic overlap between ASD phenomenology and mental health problems [11], an evaluation of the associations between ASD phenomenology and the broader behavioural phenotype in PMS may prove useful. Therefore, this study has the following aims:

i) To describe the behavioural phenotype of PMS, specifically the profile of overactivity/impulsivity, mood and repetitive behaviour. This will be achieved by comparing a sample with PMS to matched comparison groups with fragile X syndrome, Down syndrome and idiopathic ASD.
ii) To delineate the prevalence of ASD behaviours, as measured by an ASD screening tool, in PMS in comparison to matched samples with fragile X syndrome, Down syndrome and idiopathic ASD.
iii) To delineate the profile of ASD phenomenology in PMS, through analysis of subscales and items on the ASD screening tool, in comparison to matched samples with fragile X syndrome, Down syndrome and idiopathic ASD.
iv) To investigate whether individuals with PMS attain scores above the threshold for ASD on an ASD screening measure for the same reasons as matched individuals with idiopathic ASD.
v) To investigate associations between scores on the ASD screening measure and the profile of repetitive behaviour, impulsivity/overactivity and mood in individuals with PMS, compared to the matched samples with fragile X syndrome, Down syndrome and idiopathic ASD.

## Methods

### Recruitment

Participants with PMS were contacted via UNIQUE, the UK syndrome support group for rare genetic disorders, and were invited to participate in the study. Eighty-five parents and carers were contacted, and 36 completed and returned the questionnaires (return rate 42%).

Participants for the comparison groups with idiopathic ASD, fragile X syndrome and Down syndrome were recruited via the National Autistic Society, Fragile X Society and the Down’s Syndrome Association respectively. Two hundred eighty-eight carers of individuals with ASD (return rate 19.63%), 144 carers of individuals with Down syndrome (return rate 28.80%) and 212 carers of boys with fragile X syndrome (return rate 44%) completed the questionnaire pack.

### Procedure

All carers received an information sheet, cover letter, consent form, demographic questionnaire and questionnaire pack. To avoid priming, the study was described as “Understanding behaviour in people with neurodevelopmental disorders”. Carers returned the completed questionnaires and consent forms in a prepaid envelope. Ethical approval for this study was obtained from Coventry NHS Ethics Committee.

### Participants

Participants from all groups were excluded from the study if (1) they were under the age of four, as some measures were not appropriate for young children, (2) 25% or more of the data was missing or incomplete or (3) they did not have a confirmed diagnosis of the respective syndrome from an appropriate professional. For individuals with PMS, fragile X syndrome and Down syndrome, the diagnosis professionals included general practitioners, clinical geneticist, paediatricians and neurologists. For individuals with ASD, the professionals additionally included psychiatrists, clinical psychologists and educational psychologists.

Exclusions based on the above criteria resulted in a total of 30 participants with PMS. Twenty-one (70%) of the participants with PMS were diagnosed by a clinical geneticist and eight (27%) by a paediatrician. For the remaining individual with PMS (3%), diagnosis was confirmed by fluorescence in situ hybridization test.

Matched groups with ASD, fragile X syndrome and Down syndrome were then selected from the comparison samples. These groups were matched on chronological age (± 3 years) and self-help score (± 3) derived from the Wessex Scale [27]. Self-help scores were utilised as a proxy measures of degree of disability. Sixteen (53%) of the participants with fragile X syndrome were diagnosed by a paediatrician, thirteen (43%) by a clinical geneticist and one by a consultant psychiatrist. Twenty-five (83%) of the participants with Down syndrome were diagnosed by a paediatrician, one by a general practitioner (3%) and four by other professionals including during ante-natal screening (13%). Sixteen of the participants with ASD were diagnosed by a paediatrician (53%), five by a general practitioner (17%), four by a psychiatrist (13%), three by a clinical psychologist (10%), one by an educational psychologist (3%) and one by a clinical geneticist (3%).

Table 1 presents the demographic characteristics of the groups. The mean age of the total sample was 10.80 years (SD = 7.06; range = 4–39 years), 83 (69.2%) were male and 60 (50.0%) were able/partly able (score above six on the self-help subscale of the Wessex Scale). Ninety-one (75.8%) were mobile, 89 (74.2%) verbal, 100 (83.3%) had normal hearing and 94 (78.3%) had normal vision. After matching, significant differences were still found between the groups for gender (accounted for by the fact that only males with fragile X syndrome were recruited), self-help score, hearing and speech.

**Table 1:** Mean age (standard deviation) and range, percentage of males, mean self-help score (standard deviation) and percentage of participants who were mobile, verbal, had normal hearing and normal vision for all groups

		Syndrome group				Chi-square			Post hoc < .01
		PMS	ASD	FraX	DS	*df*	*χ* ^2^	*p* value	
*N*		30	30	30	30				
Age^a^	Mean (SD)	10.55 (7.08)	10.60 (7.46)	11.37 (7.02)	10.67 (7.00)	3	1.29*	.732	–
	Range	4.00–37.00	4.00–39.00	6.00–39.00	4.00–36.00				
Gender	Male (%)	13 (43.33)	26 (86.67)	30 (100.00)	14 (46.67)	3	34.19	*< .01*	ASD, FraX > PMS, DS
Self-help^b^	Mean (SD)	4.77 (1.14)	5.33 (1.24)	5.33 (1.09)	6.20 (1.06)	3	20.47*	*< .001*	DS > PMS, ASD, FraX
Mobility^b^	Fully mobile (%)	22 (73.33)	23 (76.67)	20 (66.67)	26 (86.67)	3	34.10	.33	–
Vision^b^	Normal (%)	24 (80.00)	27 (90.0)	24 (80.0)	19 (63.33)	3	6.89	.08	–
Hearing^b^	Normal (%)	26 (86.67)	27 (90.00)	29 (96.67)	18 (60.00)	3	15.23**	*.001*	PMS, ASD, FraX > DS
Speech^c^	Verbal (%)	5 (16.77)	20 (66.77)	24 (80.00)	24 (80.00)	3	33.96	*< .001*	ASD, DS, FraX > PMS

Significant differences are highlighted in italics

Groups: *PMS* Phelan–McDermid syndrome, *ASD* autism spectrum disorder, *FraX* fragile X syndrome, *DS* Down syndrome

*Kruskal–Wallis test for continuous non-normally distributed data

**Fisher’s exact test calculated

^a^In years (decimal)

^b^Data derived from the Wessex Scale

^c^According to item 1 on the SCQ “Is he/she now able to talk using short phrases or sentences”

#### Idiopathic ASD comparison group

To confirm the validity of the idiopathic ASD comparison sample as a reference group, SCQ data were compared to that of the normative sample reported in the SCQ manual [28, 29]. This method for validating an ASD reference group has been utilised previously in a study investigating the profile of autism phenomenology in genetic syndromes [24]. The manual reports the percentage of individuals in the SCQ normative sample who displayed “impairments” for each item. Data were extracted based on calculations from these percentages and the total sample size. These data were then used to calculate odds ratios at item level, using 99% confidence intervals. Odds ratio analyses revealed no significant differences between the idiopathic ASD comparison sample in the present study and the normative SCQ sample on 34 of 39 items. The idiopathic ASD comparison group in the present study was more likely to score as “impaired” on four SCQ items including three algorithm items: social chat, neologisms and unusual sensory interests, and one non-algorithm item: unusual attachments to objects. The idiopathic ASD comparison sample in the present study was less likely to score as “impaired” on seeking to share enjoyment. Overall, these findings validate the matched sample selected in this study, demonstrating that they are very similar to the normative sample reported in the SCQ.

### Measures

The questionnaire pack included the following informant-based questionnaire measures which are all appropriate for children and adults with intellectual disabilities. The order of the measures in the questionnaire pack was counterbalanced across the group to reduce order effects.

A demographic questionnaire that required information on date of birth, gender, mobility, verbal ability and diagnosis was included. The Wessex [27] was used to assess ability as a proxy IQ measure. This measure was selected as in samples of individuals with ASD and intellectual disability, adaptive functioning and IQ scores are well correlated and IQ is a significant predictor of adaptive functioning [30–32]. The Wessex comprises five subscales including continence, mobility, self-help skills, speech and literacy. For this study, the self-help subscale was used to estimate degree of ability and responses to items on mobility and vision and hearing were used to further describe the groups. The Wessex Scale has a modest inter-rater reliability at subscale level for both children and adults (mean kappa value of .62 and .54 for overall classification and item level reliability respectively; [27, 33]). The Wessex is an effective tool for large-scale questionnaire studies [33].

The Mood, Interest and Pleasure Questionnaire-Short form (MIPQ-S) [34] was used to assess mood and comprises 12 items, forming two subscales: Mood and Interest and Pleasure. The measure has good internal consistency (Cronbach’s alpha coefficients: total = .88, Mood = .79, Interest and Pleasure = .87), test-retest (.97) and inter-rater reliability (.85). Internal consistency for subscales is good (alpha coefficient range for subscales .84–.94). Concurrent validity between the MIPQ and the Aberrant Behavior Checklists (ABC) ranged from medium to strong (0.36–0.73; *p* < .001).

The Activity Questionnaire (TAQ) [35] was included to assess behaviours indicative of overactivity and impulsivity. The measure has 18 items which form three subscales of Overactivity, Impulsivity and Impulsive Speech. Item-level inter-rater reliability ranges from .31 to .75 (mean .56), and test-retest reliability ranges from .60 to .90 (mean .75). Inter-rater and test-retest reliability indices for subscales and total score exceed .70. Internal consistency for the subscales is good (alpha coefficient range for subscales .67–.94).

The Repetitive Behaviour Questionnaire (RBQ) [36] was used to assess repetitive behaviours and comprises five subscales: Stereotyped behaviour, Compulsive behaviour, Insistence on Sameness, Restricted Preferences and Repetitive Speech. Previous examination of the psychometric properties of the RBQ [36] reveals good inter-rater reliability coefficients (range .46–.80), test-retest reliability (range .61–.93; [36]) and internal consistency (alpha coefficient range for subscales .50–.78). Concurrent validity and content validity between the RBQ and the repetitive behaviour subscale of the ASQ is good (0.6; *p* < .001).

The Social Communication Questionnaire—Lifetime version [28] was included to assess ASD behaviours. The SCQ was developed as a tool for screening for ASD in children and adults and is based on the ADI-R [20]. The measure consists of 40 items which are scored to indicate the presence (a score of 1) or absence (a score of 0) of autistic impairments. These items are grouped into three subscales which correspond to impairments in communication, social interaction and repetitive and stereotyped patterns of behaviours. The authors identify a cutoff score of 15 as indicative of autistic spectrum disorder and a higher cutoff of 22 to differentiate between individuals with autism and those with other pervasive developmental disorders. The SCQ shows good concurrent validity with the ADI-R and the ADOS [37]. Importantly, the SCQ demonstrates higher precision in samples with low IQ than other screening tools, including the Children’s Communication Checklist and the Social Responsiveness Scale [38]. Internal consistency is also good (*α* = .90 for the total scale). The SCQ has good item-level validity, with 33 out of 39 items differentiating between those with ASD and those without ASD [29]. The fragile X and Down syndrome groups completed an earlier version of the SCQ (Autism Screening Questionnaire (ASQ)). One item differed between the ASQ and SCQ for non-verbal individuals for subscale scoring (item 20, social chat). Following the approach taken by Moss and colleagues [24] to ensure consistency across the groups, this item was treated as missing and pro-rated for all non-verbal participants. The prorated score was calculated as the mean item score, based on other completed items within the communication domain. Item 20 was not included in the item-level analysis.

### Data analysis

Data were tested for normality using Kolmogorov–Smirnov tests. Where data were not normally distributed (*p* < .05), non-parametric techniques were employed. To control for multiple comparisons, alpha levels were set at a conservative value of *p* < .01.

## Results

### Behavioural phenotype of PMS

In order to fulfil the first aim of the study, delineating the behavioural phenotype of PMS, compared to the matched contrast groups, subscale scores were derived to describe mood (taken from the MIPQ), activity levels (taken from the TAQ) and repetitive behaviour (taken from the RBQ). A series of Kruskal–Wallis tests were performed to test for differences in the subscales between the groups. Table 2 displays the subscale, total scores and Kruskal–Wallis statistics.

**Table 2:** MIPQ, RBQ and TAQ subscale/total score medians and interquartile ranges (excluding verbal subscales)

Measure	Median scores (interquartile range)				Kruskal–Wallis test			Post hoc < .01
	PMS	FraX	DS	Idiopathic ASD	df	K	*p* value^a^	
*MIPQ-S*								
Mood	20.00 (17.75–21.25)	21.00 (19.75–21.18)	22.00 (19.75–22.25)	17.00 (16.00–21.00)	3	22.26	*< .001*	FraX, DS > ASD
Interest and Pleasure	16.00 (12.88–20.00)	18.00 (13.00–19.25)	20.00 (17.75–22.00)	12.00 (8.75–15.25)	3	27.53	*< .001*	DS > ASD
Total score	36.00 (31.75–41.00)	39.00 (31.75–42.00)	41.00 (38.75–44.00)	29.50 (25.00–35.25)	3	30.34	*< .001*	PMS, DS, FraX > ASD, DS > PMS
*RBQ*								
Stereotyped behaviour	7.50 (5.75–12.00)	9.00 (7.37–12.00)	0.50 (0.00–6.50)	9.50 (6.00–12.00)	3	24.84	*< .001*	PMS, ASD, FraX > DS
Compulsive behaviour	0.00 (0.00–4.50)	6.00 (0.00–9.00)	0.00 (0.00–3.25)	6.00 (3.50–15.25)	3	21.81	*< .001*	ASD > DS, PMS
Insistence on sameness	0.00 (0.00–2.50)	4.00 (3.00–7.25)	0.00 (0.00–2.25)	4.00 (2.00–6.00)	3	30.45	*< .001*	ASD, FRaX > DS, PMS
Total score	12.00 (7.75–19.75)	29.50 (22.50–36.25)	10.50 (4.00–15.25)	25.00 (16.00–32.50)	3	39.44	*< .001*	ASD, FRaX > DS, PMS
*TAQ*								
Impulsivity	16.50 (12.00–20.25)	20.72 (15.75–23.25)	12.00 (7.75–18.25)	20.00 (16.50–23.00)	3	18.22	*< .001*	ASD, FraX > DS
Overactivity	19.00 (12.75–25.25)	24.00 (12.75–32)	9.50 (6.00–23.25)	20.50 (15.75–30.00)	3	14.54	*.002*	ASD, FraX > DS
Total score	37.00 (26.50–45.25)	48.50 (32.00–59.25)	23.00 (17.00–41.75)	50.00 (33.25–53.75)	3	17.45	*.001*	ASD, FraX > DS

^a^Significant differences (*p* < .01) are indicated in italics

The results in Table 2 reveal that individuals with PMS had significantly higher total mood scores than individuals with idiopathic ASD,^1^ although they also demonstrated significantly lower total mood scores than individuals with Down syndrome. The PMS group evidenced significantly higher levels of stereotyped behaviour than individuals with Down syndrome. However, they also had significantly lower scores for compulsive behaviour than the idiopathic ASD group. Additionally, individuals with PMS obtained significantly lower scores for insistence on sameness and total repetitive behaviour than both the fragile X and idiopathic ASD groups. Individuals with PMS did not differ from individuals with idiopathic ASD, fragile X or Down syndrome on measures of activity level.

In summary, individuals with PMS evidenced higher mood, but lower levels of repetitive behaviour than those with idiopathic ASD. The PMS group had lower mood scores than those with Down syndrome. The activity levels in individuals with PMS did not differ to those identified in any of the contrast groups.

### Prevalence of ASD phenomenology in PMS

In order to investigate the second aim of the study, to delineate the prevalence of ASD behaviours in PMS in comparison to the contrast groups, the percentage of each group scoring above the cutoff for ASD (score ≥ 15) and autism (score ≥ 22) were derived from the SCQ. These prevalence data were compared between groups using chi-square tests. Table 3 displays the results.

**Table 3:** Percentage scoring above the ASD and autism cutoffs on the SCQ

Group	% scoring above ASD cutoff (N)	% scoring above autism cutoff (N)
PMS	86.7 (26)	56.7 (17)
FraX	80.0 (24)	51.9 (14)
DS	23.3 (7)	22.2 (6)
Idiopathic ASD	100.0 (30)	76.7 (23)

The results revealed that 86.7% of individuals with PMS scored above the threshold for ASD and 56.7% scored above the threshold for autism. There was a significant difference between the proportion of individuals in each group scoring above the cutoff for ASD (*χ* ^2^ (3) = 51.38, *p* < .001; ASD, FraX, PMS > DS). There was also a significant difference between the proportion of individuals in each group scoring above the cutoff for autism (*χ* ^2^ (3) = 17.17, *p* = .001; ASD, FraX, PMS > DS).

In summary, the proportion of individuals with PMS who scored above the SCQ thresholds for ASD and autism was higher than those in the Down syndrome group, but did not differ from those with idiopathic ASD or fragile X syndrome.

### Profile of ASD phenomenology in PMS

In order to investigate the third aim of the study, to delineate the profile of ASD phenomenology in PMS, subscale scores for Communication, Repetitive Behaviour and Reciprocal Social Interaction domains were derived from the SCQ for the PMS group and the matched comparison groups. In order to allow for the high proportion of individuals with PMS who were non-verbal, subscale scores excluding verbal items were generated and differences between the groups were evaluated using Kruskal–Wallis tests. These subscale and total scores are presented in Table 4 with Kruskal–Wallis test results to evaluate differences between the groups.

**Table 4:** SCQ subscale/total score medians and interquartile ranges, calculated with and without verbal items

Domain	Median scores all items (interquartile range)				Kruskal–Wallis test			Post hoc < .01
	PMS	FraX	DS	Idiopathic ASD	df	k	*p* value^a^	
Communication (verbal items removed)	7.00 (4.00–7.00)	4.00 (3.00–5.80)	1.00 (0.00–4.00)	6.00 (4.00–7.00)	3	29.97	< .001	ASD, PMS > DS
Repetitive Behaviour (verbal items removed)	4.00 (2.75–5.00)	5.00 (3.00–6.00)	1.00 (0.00–4.00)	5.50 (4.00–7.00)	3	32.72	*< .001*	ASD, FraX > DS
Reciprocal Social Interaction	10.00 (7.75–13.00)	9.00 (6.00–11.00)	3.00 (1.00–8.00)	11.25 (9.00–13.00)	3	28.04	*< .001*	ASD, PMS > DS
Total score (verbal items removed)	21.50 (18.75–25.00)	18.50 (12.00–23.25)	5.00 (2.00–12.50)	23.40 (19.00–27.00)	3	35.66	*< .001*	ASD, PMS > DS

^a^Significant differences (*p* < .01) are indicated in italics

The results in Table 4 reveal that the PMS group showed significantly more “ASD-like” communication impairments than the Down syndrome group, but did not differ from any of the groups in repetitive behaviours. The PMS group evidenced significantly more “ASD-like” reciprocal social interaction impairments than individuals with Down syndrome. At total score level, the PMS group were significantly more impaired than those with Down syndrome.

In order to further evaluate the profile of ASD phenomenology in PMS, the percentage of individuals in each group scoring as “impaired” (score of 1) for each non-verbal item of the SCQ was calculated. Differences between the groups for each item were evaluated using chi-square tests. Table 5 presents the results.

**Table 5:** Percentage of individuals that scored as “impaired” on SCQ non-verbal algorithm items

		% impairment				Chi-square				
Domain	Item	PMS	FraX	DS	ASD	*df*	χ^2^	*p* value^a^	Post hoc < .01	
Communication	Imitation	76.7	46.7	33.3	83.3	3	*15.53*	*.001*	ASD > FraX, DS; PMS > DS	+^b^
	Pointing	86.7	56.7	33.3	70.0	3	*15.87*	*.001*	PMS > DS	+
	Gestures	70.0	46.7	36.7	60.0	3	5.53	.137	N/A	N/A
	Nodding to mean *yes*	86.7	46.7	23.3	83.3	3	*31.50*	*< .001*	ASD, PMS > FraX, DS	+ +
	Head shaking to mean *no*	73.3	36.7	23.3	76.7	3	*21.22*	*< .001*	ASD > FraX, DS; PMS > DS	+
	Imitative social play	80.0	60.0	16.7	76.7	3	*28.22*	*< .001*	ASD, PMS, FraX > DS	+
	Imaginative play	83.3	80.0	36.7	80.0	3	*21.05*	*< .001*	ASD, PMS, FraX > DS	+
Repetitive Behaviour	Rituals	40.0	73.3	46.7	83.3	3	*17.18*	*.001*	ASD > PMS, DS; FraX > PMS	− −^c^
	Unusual preoccupations	60.0	63.3	20.0	70.0	3	*19.44*	*< .001*	ASD, PMS, FraX > DS	+
	Stereotyped play	66.7	60.0	30.0	76.7	3	*13.97*	*.003*	ASD, PMS > DS	+
	Circumscribed interests	30.0	56.7	36.7	60.0	3	7.40	.060	N/A	N/A
	Sensory interests	53.3	43.3	13.3	83.3	3	*29.15*	*< .001*	ASD > FRaX, DS; PMS > DS	+
	Hand stereotypies	70.0	86.7	33.3	90.0	3	*27.40*	*< .001*	ASD, PMS, FRaX > DS	+
	Body stereotypies	56.7	60.0	23.3	66.7	3	*12.64*	*.005*	ASD, FraX > DS	N/A
Reciprocal Social Interaction	Inappropriate facial expressions	40.0	23.3	6.7	40.0	3	*11.49*	*.009*	ASD, PMS > DS	*+*
	Use of other’s body to communicate	83.3	56.7	40.0	86.7	3	*20.10*	*< .001*	ASD, PMS > DS	*+*
	Friends	70.0	80.0	30.0	76.7	3	*19.72*	*< .001*	ASD, PMS, FRaX > DS	+
	Eye contact	56.7	53.3	23.3	56.7	3	8.57	.036	N/A	N/A
	Social smiling	40.0	36.7	16.7	66.7	3	*13.66*	*.003*	ASD > DS	N/A
	Showing and directing attention	70.0	46.7	16.7	36.7	3	*15.69*	*.001*	PMS > ASD, DS	+ +
	Offering to share	80.0	63.3	33.3	76.7	3	*15.41*	*.001*	ASD, PMS > DS	+
	Seeking to share enjoyment	43.3	26.7	23.3	36.7	3	2.27	.519	N/A	N/A
	Offering comfort	83.3	56.7	20.0	86.7	3	*32.27*	*< .001*	ASD, PMS, FraX > DS	+
	Quality of social overtures	56.7	26.7	13.3	46.7	3	*12.67*	*.005*	ASD, PMS > DS	+
	Range of facial expression	63.3	56.7	23.3	80.0	3	*17.82*	*< .001*	ASD, PMS, FRaX > DS	+
	Interest in children	60.0	56.7	26.7	90.0	3	*21.91*	*< .001*	ASD > PMS, DS	–
	Response to other children’s approaches	53.3	63.3	23.3	86.7	3	*23.64*	*< .001*	ASD > PMS, DS; FraX > DS	–
	Imaginative play with peers	90.0	86.7	60.0	100.0	3	*14.12* ^d^	*.001*	ASD > DS	N/A
	Group play	86.7	73.3	46.7	86.7	3	*12.64*	*.005*	ASD, PMS > DS	+

^a^Significant differences are highlighted in italics (*p* < .01)

^b^“+” indicates that significantly more individuals in the PMS group scored as impaired one of the comparison groups

^c^“−” indicates significantly fewer individuals in the PMS group scored as impaired than one of the comparison groups

^d^Fisher’s exact test calculated

The results revealed that significantly more of the PMS group than the Down syndrome group scored as impaired on five of the seven items in the Communication subscale. Additionally, for the item describing “nodding to say *no*”, significantly more individuals with PMS were identified as impaired than individuals with fragile X syndrome and Down syndrome. Significantly more of the PMS group than the Down syndrome group scored as impaired on four of the seven items in the Repetitive Behaviour subscale. However, significantly fewer individuals with PMS were identified as showing ritualistic repetitive behaviours, relative to individuals with idiopathic ASD and fragile X syndrome. Significantly more of the PMS group than the Down syndrome group scored as impaired on eight of the 15 items in the Reciprocal Social Interaction subscale. Importantly, significantly more individuals with PMS showed impairments in “showing and directing attention” than individuals with idiopathic ASD or Down syndrome. Conversely, significantly fewer individuals with PMS showed impairments in items regarding interest in other children, and responding to other children’s approaches, than individuals with idiopathic ASD.

In summary, the PMS group did not differ from the idiopathic ASD or fragile X syndrome groups in levels of “ASD-like” communication impairments. When verbal items were removed, they evidenced significantly more communication impairments than those with Down syndrome. At the item level, individuals with PMS evidenced specific impairments in using nodding to communicate with others. The PMS group did not differ from the fragile X or Down syndrome groups in levels of “ASD-like” repetitive behaviour but did evidence significantly less impairment than the idiopathic ASD group when verbal items were included in analysis. At the item level, the PMS group demonstrated significantly less ritualistic behaviour. The PMS group evidenced significantly more impairment in social interaction than the Down syndrome group and did not differ from the idiopathic ASD or fragile X syndrome groups. At the item level, those with PMS evidenced significant impairment in showing and directing attention, but relative preservation of interest in, and responses to, other children compared to those with idiopathic ASD.

### Analysis of items associated with meeting threshold for autism in PMS

In order to meet the fourth aim of the study, to explore whether individuals with PMS reach threshold on the SCQ for similar reasons as individuals with idiopathic ASD, item-level comparisons were conducted, comparing those with PMS who scored over the threshold for autism (≥ 22) to those with idiopathic ASD who also scored over the threshold for autism. The number of individuals in the PMS group scoring as “impaired” on each item was compared to the number of individuals in the idiopathic ASD comparison group scoring as impaired on each item, using relative risk ratios with 99% confidence intervals.

**Fig. 1:**
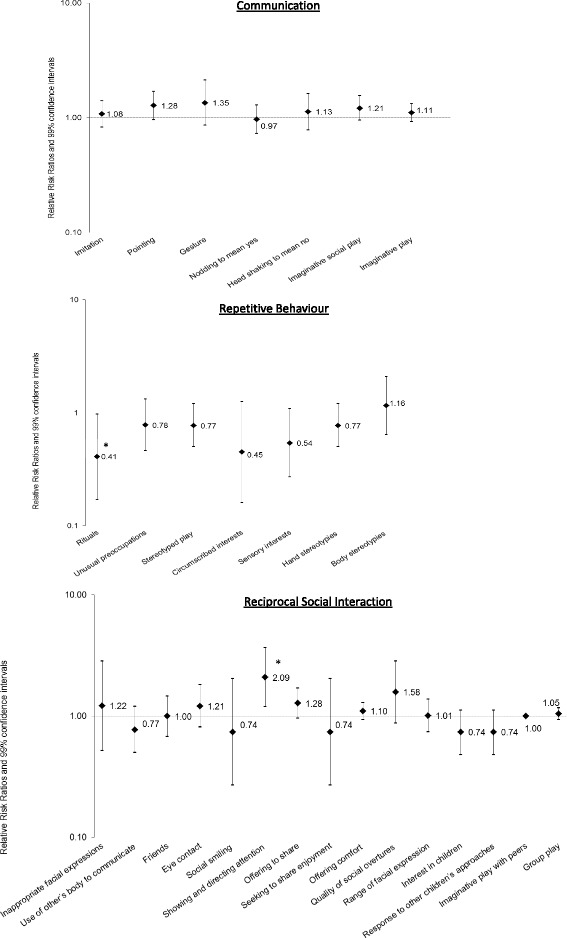
Relative risk ratios comparing the PMS and idiopathic ASD groups on SCQ items in domain algorithms. Odds ratios with 99% confidence intervals for SCQ items comparing individuals with PMS who score over the autism threshold to the idiopathic ASD group. The asterisk indicates significant difference. The *Y*-axis scales differ between subscales

The results in Fig. 1 reveal that individuals with PMS who met criteria for autism on the SCQ were no more or less likely to evidence impairments in the Communication items than individuals with idiopathic ASD. However, they were significantly more likely to score as impaired on the “Showing and directing attention” item in the Reciprocal Social Interaction domain and significantly less likely to score as impaired on the “Rituals” item in the Repetitive Behaviour domain.

### Association between behavioural phenotype and ASD phenomenology in PMS

In order to fulfil the final aim of the study, to investigate the association between ASD phenomenology and behavioural phenotype, a series of Spearman’s rank correlations were conducted for each group, evaluating associations between total SCQ score and demographic characteristics (self-help score and chronological age) and behavioural characteristics (mood, activity and repetitive behaviour).

Table 6 reveals that higher scores on the SCQ were significantly correlated with lower scores on the interest and pleasure subscale for individuals with PMS. The correlation between SCQ score and mood score approached significance (*r*_s_ (28) = − .37, *p* = .043).

**Table 6:** Correlation coefficients for Spearman’s rank correlations between total SCQ score and demographic/behavioural characteristics

Demographic/behavioural characteristic	PMS	FraX	DS	Idiopathic ASD
Self-help	− 0.20	− 0.07	− 0.21	− 0.02
Age	0.35	0.28	0.24	− 0.11
Mood	− 0.28	− 0.06	0.07	− 0.40
Interest and pleasure	*− 0.50* ^a^	− 0.20	− 0.38	− 0.14
Stereotyped behaviour	0.36	0.34	*0.63*	0.12
Compulsive behaviour	− 0.08	− 0.21	0.22	0.39
Insistence on sameness	− 0.04	− 0.32	0.13	0.04
Impulsivity	0.21	0.22	0.32	0.25
Overactivity	0.32	0.25	0.19	0.23

^a^Significant correlations (*p* < .01) are highlighted in italics

## Discussion

The behavioural characteristics, prevalence and profile of ASD phenomenology in PMS were delineated in this study. The association between ASD phenomenology and broader behavioural and demographic characteristics was also evaluated. The novel recruitment of comparison groups with fragile X and Down syndrome, in which the profile of ASD phenomenology is well described, strengthens the validity of the study. The inclusion of a matched idiopathic ASD comparison group allows for robust delineation of the profile of ASD phenomenology in PMS. The use of validated measures, with appropriate psychometric properties established in populations with intellectual disabilities further contributes to the validity and reliability of the findings.

The results of the behavioural phenotype analyses revealed that individuals with PMS evidenced higher total mood scores than those with idiopathic ASD, but lower total mood scores than those with Down syndrome. Despite the between group differences identified at the total score level, there were no identified differences on the Mood or Interest and Pleasure subscales between the PMS and comparison groups. These findings support previous research identifying low mood in individuals with PMS [11], but also demonstrate the utility of including multiple comparison groups in order to position the behavioural phenotype in PMS relative to other syndromes. The PMS group achieved higher total mood scores than those with idiopathic ASD and comparable total mood scores to those with fragile X syndrome, suggesting that whilst lower mood is present in PMS, it may not be significantly atypical, given the degree of intellectual disability in the group.

The use of a carefully designed and detailed assessment of repetitive behaviour [36] revealed a mixed profile in individuals with PMS. The group evidenced similar levels of stereotyped behaviour, but lower levels of compulsive behaviour, insistence on sameness and total repetitive behaviour than both the fragile X syndrome and idiopathic ASD groups. This finding supports and synthesises divergent results demonstrating low levels of repetitive behaviour in PMS [26] and the presence of repetitive and sensory-motor behaviours in the group [14]. Individuals with PMS appear to evidence a dissociation between motor-driven repetitive behaviours, which are common in the sample, and more compulsive and routine-driven behaviours, which are less evident in the group. It is important to note that this finding is at the level of the total sample, including those who meet threshold for autism and those who do not. Finally, the results revealed no significant differences in levels of overactivity or impulsivity between the PMS and comparison groups. This finding differs from those previously reported, where high levels of ADHD-type behaviours were identified [11, 12]. However, previous research did not compare individuals with PMS to matched comparison groups, and thus, the high levels of activity and impulsivity may be more appropriately associated with the degree of intellectual disability in PMS rather than the behavioural phenotype of PMS per se.

The results demonstrated a high prevalence of ASD phenomenology in PMS, with 87% meeting threshold for ASD and 57% meeting the more stringent criteria for autism. These findings support the prevalence figures identified in previous studies using screening measures (94% mild–moderate ASD, 67% severe ASD [2]; 85% ASD, 67% autism [12]) and diagnostic tools (84% ASD, 75% autistic disorder [14]). The results of this study extend findings by demonstrating that a similar proportion of individuals with PMS meet threshold for ASD and autism as males with fragile X syndrome, in whom ASD phenomenology is common. Importantly, the proportion of individuals in the PMS group meeting clinical thresholds on the SCQ was significantly higher than the Down syndrome group, suggesting that a high prevalence of ASD phenomenology can be associated with the behavioural phenotype of PMS. It is important to note that whilst this study has demonstrated a high prevalence of ASD phenomenology in PMS, this does not directly equate to a high prevalence of ASD diagnoses in PMS, given the necessity of thorough, multimodal assessment in the clinical diagnoses of ASD.

Analyses to evaluate the profile of ASD phenomenology in the total PMS sample provided heterogeneous results across impairments in communication, social interaction and repetitive and restricted behaviour. Firstly, at subscale level, the group did not differ from the idiopathic ASD or fragile X syndrome groups in “ASD-like” communication impairments. Additionally, the PMS group evidenced more impairments than those with Down syndrome. This finding supports previous results highlighting “ASD-like” impairments in communication in PMS [14, 26]. Item-level analyses extended these findings to reveal that the PMS group evidenced specific impairments in “nodding to communicate *yes*”, with a higher proportion of the PMS sample scoring as impaired on this item than all three comparison groups, although this did not reach statistical significance when compared to the idiopathic ASD group who scored over the autism threshold. The PMS group did not significantly differ from the idiopathic ASD group on any item in the communication domain, suggesting that the profile of “ASD-like” communication impairments is similar in that of total PMS and idiopathic ASD groups.

Secondly, the PMS group did not differ from the idiopathic ASD, fragile X or Down syndrome groups in “ASD-like” repetitive behaviours. However, item-level analysis revealed that the PMS group was significantly less likely to engage in non-verbal ritualistic behaviours than those with fragile X syndrome or idiopathic ASD. This difference remained significant in the secondary analysis of individuals with PMS who scored above the clinical threshold for autism. Thus, the profile of repetitive behaviour is still somewhat unclear in PMS. Fine-grained observational analysis of repetitive behaviours would be beneficial, in order to detail topography, frequency and any potential management difficulties of repetitive behaviour in the syndrome.

Finally, at the subscale level, the PMS group evidenced significantly more impairments in social interaction than the Down syndrome group and showed comparable levels of impairment to the idiopathic ASD and fragile X syndrome groups. This finding supports data demonstrating “ASD-like” social interaction impairments in PMS [14, 26]. An interesting dissociation in social interaction was revealed at item level; the PMS group showed significantly more impairments in “Showing and directing attention” than both the Down syndrome and idiopathic ASD groups, but significantly less impairment in items assessing interest in, and responses to, other children. One interpretation of this finding is that there is a divergence in social skills and social motivation in PMS, with relatively preserved social motivation in contrast to deficits in social competence, potentially due to low levels of expressive speech. Alternatively, the result may represent a specific impairment in initiating interaction, with relatively preserved abilities to respond to interactions initiated by others. This finding warrants further investigation, including attempts to replicate the results in larger samples with PMS, using both indirect and direct assessments of social competence and motivation.

Whilst the profile of ASD impairments was varied within the total PMS sample, the results within the subgroup that scored above the autism threshold were very similar to the profile of behaviour in the idiopathic ASD group, suggesting that both groups reach clinical thresholds for autism due to a similar profile of behaviours. The results in this subgroup revealed that individuals with PMS were neither more nor less likely to score on items in the communication, social interaction or repetitive behaviour domain, than those with idiopathic ASD except for an increased impairment in “Showing and directing attention” and a decreased likelihood of ritualistic behaviour. This finding extends previous research, affording a more refined understanding of the nature ASD impairments in affected individuals with PMS. The result suggests that when individuals with PMS meet criteria for autism, they do so for similar reasons to those with idiopathic ASD. Clinically, this may indicate that interventions to support individuals with idiopathic ASD could be usefully applied to individuals with PMS who meet the diagnostic criteria. The result also replicates the specific deficit noted in the total sample in showing and directing attention. Interventions to extend the behavioural repertoires of individuals with PMS focused on behaviour to recruit and maintain others’ attention which may be warranted in this population.

The final results of this study demonstrated that across all demographic and behavioural scores, only “Interest and pleasure” was (negatively) correlated with SCQ score in the PMS group. The correlation between “Mood” and total SCQ score approached significance. These findings lend tangential support to previous research indicating an association between the presentation of mood disorders and ASD phenomenology in the syndrome [11]. However, given the strength of evidence of behaviours indicative of ASD in PMS, the correlation between interest and pleasure and SCQ score is not interpreted as substantiation of mood disorders being wholly explanatory for ASD phenomenology in PMS. Instead, it is possible that behaviours indicative of low mood are associated with ASD impairments in PMS. Alternatively, it may be that mood disorders and ASD impairments co-exist within PMS due to similar genetic underpinnings, perhaps with greater severity of mood disorder being associated with more significant genetic deletion, as ASD phenomenology is hypothesised to [8]. These hypotheses are tentative and further research is required to delineate the association between mood and ASD phenomenology in PMS, including any causal links between the two phenomena.

A number of caveats must be considered when interpreting the findings in this study. Firstly, the assessment of ASD phenomenology is somewhat limited, due to the utilisation of a screening measure rather than a diagnostic measure; the “gold standard” for assessment of ASD in individuals with intellectual disability is a combination of ADOS and ADI-R. However, utilising a brief parent screening measure reduced time and assessment demands and conferred the advantage of assessing multiple comparison groups in order to position the profile of ASD phenomenology in PMS relative to other syndromes [39]. Additionally, the SCQ is recognised as more appropriate for assessing ASD phenomenology in samples with intellectual disabilities than other ASD screening tools [38]. Similarly, the Wessex adaptive behaviour scores were utilised as a proxy measure for intellectual disability. Whilst it would have been beneficial to conduct full cognitive assessments of all of the participants, it would not have been possible within the scope of this study. Thus, a brief assessment of adaptive behaviour was chosen in order to balance the need to assess intellectual disability and the need to maximise participants in all the four groups. The limitations imposed by this method of assessing adaptive functioning should be considered as a caveat to the present study, particularly as the Wessex score was used as one of the indices for matching the groups. Future studies should seek to include robust cognitive assessments of intellectual functioning. Secondly, despite careful matching of the groups, it was not possible to reduce all differences in adaptive behaviour. Therefore, the Down syndrome group were significantly more able than the PMS, fragile X syndrome and idiopathic ASD samples. Previous researchers have argued that delineating the behavioural phenotype of a given genetic syndrome in relation to multiple other syndromes reduces the need for chronological or mental age matched comparison groups [39]. Additionally, the PMS, fragile X syndrome and idiopathic ASD groups were well matched for chronological age and adaptive ability. Nonetheless, the results should be interpreted with this caveat in mind. Finally, due to the relatively small PMS sample, there was insufficient statistical power to test causal associations between expressive speech, adaptive behaviour and ASD scores. Previous research has highlighted that it is important to explore these associations in samples with genetic syndromes [22]. Correlational evidence from this study indicates that adaptive behaviour was not associated with SCQ score; however, this still warrants further exploration in larger sample sizes, where causal statistical modelling is possible.

The results of this study have a number of important clinical implications. The results indicate that assessment of behaviours indicative of low mood should be routine in individuals with PMS. Research in individuals with severe intellectual disabilities has revealed that low mood scores may indicate pain and undiagnosed health conditions [40–42]. There are reports of gastro-oesophageal reflux and other painful conditions in PMS [1, 15]. Therefore, thorough health assessments should routinely be conducted for individuals with PMS. The results of this study have implications for research investigating the genetic underpinnings of idiopathic ASD. The results demonstrate that those with high levels of ASD impairment evidence a profile of ASD impairments that is similar to that of individuals with idiopathic ASD. However, the wider PMS sample presents a more atypical pattern with fewer impairments in repetitive behaviours. This may suggest that social and communicative impairments would be a useful autism endophenotype to be investigated in relation to 22q13.3 deletions and *SHANK3* mutations more broadly [16]. Finally, the results of this study have implications for clinical trials in PMS. To translate recent pharmacological successes from pre-clinical studies to human trials [43, 44], nuanced behavioural phenotyping and identification of measures sensitive to change in behavioural characteristics are required. This study has identified unique aspects of the behavioural phenotype of PMS which should be considered as potential clinical targets (low mood, autism spectrum disorder characteristics) using measures appropriate for the level of intellectual disability present in the syndrome, highlighting both targets for intervention and measures with sensitivity to detect these difficulties.

In summary, this study has demonstrated that differences in mood and repetitive behaviour are common in PMS. Additionally, autism spectrum disorder phenomenology is prevalent within the syndrome. The profile of ASD impairments in the total sample with PMS is heterogeneous; the profile within those who meet clinical threshold for autism is analogous to those with idiopathic ASD. The presence of ASD phenomenology is associated with lower mood in those with PMS.

## Conclusions

The results of this study demonstrate that ASD phenomenology is common in PMS and can be considered to be a component of the behavioural phenotype. These data support the assertion that *SHANK3* mutations may be causally implicated in the development of ASD-type behaviours, particularly social and communication deficits, although further research is required to delineate the specificity of this endophenotype. The results extended previous studies by demonstrating that whilst the profile of ASD phenomenology in PMS is atypical, the profile in those who score above threshold on an autism screening measure is analogous to idiopathic ASD. This suggests that ASD-specific interventions could be usefully applied to groups with PMS who meet criteria for ASD. Fine-grained analysis of ASD phenomenology revealed an emerging dissociation between deficits in behaviours indicative of social skill, but relative preservation in behaviours indicative of social motivation. This suggests potential targets for psychological interventions in PMS.

## Abbreviations

ADHD: Attention deficit hyperactivity disorder
ADI-R: Autism Diagnostic Interview-Revised
ADOS: Autism Diagnostic Observation Schedule
ASD: Autism spectrum disorder
ASQ: Autism Screening Questionnaire
DS: Down syndrome
FraX: Fragile X syndrome
I_h_: Hyperpolarization-activated cation
MIPQ-S: The Mood Interest and Pleasure Questionnaire-Short form
PMS: Phelan–McDermid syndrome
RBQ: The Repetitive Behaviour Questionnaire
SCQ: Social Communication Questionnaire
TAQ: The Activity Questionnaire

## Footnotes

1 For brevity and to prevent duplication of results from previously published data, this manuscript will only describe the differences between the PMS group and other comparison groups, rather than also describing inter-comparison group differences.
